# Clinicopathological Features as Prognostic Predictors of Poor Outcome in Papillary Thyroid Carcinoma

**DOI:** 10.3390/cancers12113186

**Published:** 2020-10-29

**Authors:** Antónia Afonso Póvoa, Elisabete Teixeira, Maria Rosa Bella-Cueto, Miguel Melo, Maria João Oliveira, Manuel Sobrinho-Simões, Jorge Maciel, Paula Soares

**Affiliations:** 1 Department of General Surgery, Centro Hospitalar de Vila Nova de Gaia/Espinho (CHVNG/E), 4434-502 Gaia, Portugal; jmacielbarbosa@netcabo.pt; 2 IPATIMUP-Instituto de Patologia e Imunologia Molecular, Universidade do Porto, 4200-135 Porto, Portugal; eteixeira@ipatimup.pt (E.T.); jmiguelmelo@live.com.pt (M.M.); ssimoes@ipatimup.pt (M.S.-S.); 3 Cancer Signaling and Metabolism, i3S-Instituto de Investigação e Inovação em Saúde, Universidade do Porto, 4200-135 Porto, Portugal; 4 Department of Pathology, Parc Taulí Sabadell Hospital Universitari-Institut d’Investigació i Innovació Parc Taulí-I3PT-Universitat Autònoma de Barcelona, 08208 Sabadell, Spain; rbella@tauli.cat; 5 Department of Endocrinology, Centro Hospitalar Universitário de Coimbra, 3004-561 Coimbra, Portugal; 6 Department of Endocrinology, Centro Hospitalar de Vila Nova de Gaia/Espinho (CHVNG/E), 4434-502 Gaia, Portugal; mjoaooliveira1@sapo.pt; 7 Department of Pathology, Centro Hospitalar Universitário São João, 4200-319 Porto, Portugal; 8 Departamento de Patologia, Faculdade de Medicina da Universidade do Porto, 4200-319 Porto, Portugal; 9 Faculdade de Ciências da Saúde, Universidade Fernando Pessoa, 4249-004 Porto, Portugal

**Keywords:** thyroid cancer, prognosis, clinicopatholigical, outcomes

## Abstract

**Simple Summary:**

Thyroid cancer incidence is increasing, with overdiagnosis being the major driver of the thyroid cancer “epidemic”. Papillary thyroid carcinoma, usually with excellent prognosis, sometimes has an aggressive metastatic pattern. This heterogeneity in progression makes it difficult to tailor treatment strategies for an individual patient. We aimed to identify clinicopathological factors associated with papillary thyroid carcinoma recurrence, persistence, and specific mortality. Our study supports that both pre-surgical factors, such as male gender, presence of psammoma bodies, gross extra-thyroidal extension, and lateral compartment lymph node metastases, as well as lymph vessel invasion, venous invasion, presence of necrosis, and incomplete surgical resection, should be taken into consideration regarding treatment and follow-up of PTC patients. The same is true when analysis is restricted to stage I patients. The importance of this report is to emphasize clinical and imaging pre-surgical thyroid cancer patients’ evaluation for an appropriate surgical treatment and patient prognosis.

**Abstract:**

Papillary thyroid cancer (PTC) has an indolent nature and usually excellent prognosis. Some PTC clinicopathological features may contribute to the development of aggressive metastatic disease. In this work, we want to evaluate PTC clinicopathological features that are presurgical prognostic predictors of patients’ outcomes and find which indicators are more adequate for tailoring surgical procedures and follow-up. We studied a series of 241 PTC patients submitted to surgery. All patients’ files and histological tumor samples were reviewed. The 8th edition AJCC/UICC (American Joint Committee on Cancer/Union for International Cancer) Controlstaging system and the 2015 American Thyroid Association risk stratification system were used. Total thyroidectomy was performed in 228 patients, lymphadenectomy in 28 patients. Gross extrathyroidal extension (ETE) was present in 10 patients and 31 tumor resection margins were incomplete. Cervical lymph node metastases (LNMs) were present in 34 patients and distant metastases at diagnosis in four patients. In multivariate analysis, male gender (OR = 15.4, *p* = 0.015), venous invasion (OR = 16.7, *p* = 0.022), and lateral compartment LNM (OR = 26.7, *p* = 0.004) were predictors of mortality; psammoma bodies (PBs) (OR = 4.5, *p* = 0.008), lymph vessel invasion (OR = 6.9, *p* < 0.001), and gross ETE (OR = 16.1, *p* = 0.001) were predictors of structural disease status; male gender (OR = 2.9, *p* = 0.011), lymph vessel invasion (OR = 2.8, *p* = 0.006), and incomplete resection margins (OR = 4.6, *p* < 0.001) were predictors of recurrent/persistent disease. Our study supports that the factors helping to tailor patient’s surgery are male gender, presence of PBs, gross ETE, and lateral compartment LNM. Together with pathological factors, lymph vessel invasion, venous invasion, necrosis, and incomplete surgical resection, should be taken into consideration regarding treatment and follow-up of patients.

## 1. Introduction

Thyroid cancer (TC) is the most common endocrine malignancy [1,2]. According to National Cancer Institute surveillance, epidemiology, and end results (SEER), the yearly TC incidence has nearly tripled since 1975. Almost the entire change has been attributed to an increase in the incidence of papillary thyroid microcarcinoma (mPTC) [2,3]. In 2020, TC is the fifth most common cancer in women [4] and overdiagnosis is a major driver of the TC “epidemic” [5].

PTC is the most common malignancy originating from the thyroid (80–90% of all TC types [1]). Treatment includes surgical resection of the thyroid gland with radioiodine (RAI) ablation in selected cases. PTC generally has an indolent nature and good, or even excellent, prognosis after surgery, with survival rates for adults of 92–98% at 10-year follow-up [6]. Yet, some PTC patients (5–10%) develop highly aggressive metastatic disease [7], require further treatment, have poor quality of life, and a higher mortality rate. In order to differentiate patients with a worse prognosis from patients with excellent outcomes, if possible, at a pre-surgical stage, it is essential to evaluate appropriately the clinicopathological features of PTC, in an attempt to better predict patient prognosis and offer a more adequate treatment. Many risk factors and indicators of poor prognosis, including male gender, older age, increased tumor size (≥4 cm), gross extrathyroidal extension (ETE) [8], presence of lymph node metastases (LNMs) ≥3 cm and distant metastases (DMs) [9], that independently affect disease-free survival (DFS) and disease-specific survival (DSS) [10].

In the present study, we aimed to evaluate the specific association of clinicopathological features as prognostic predictors of recurrent/persistent disease, structural disease status and disease specific mortality, in a consecutive series of PTC patients. We also aimed to find which surgical procedures are more adequate to treat and follow patients. Patients’ quality of life is of paramount importance, so it is important to aggressively treat an aggressive cancer, as is taking off the psychological burden of an indolent one. Determining which clinicopathological features are prognostic predictors of each of the established outcomes is important since they have different impacts on patients’ quality of life.

## 2. Results

A detailed presentation of data can be accessed in Table S2.

### 2.1. Demography and Histopathology 

Tumors were classified according to the WHO classification [7], and subdivided into four categories: classical PTC (*n* = 157), follicular variant PTC (*n* = 55), oncocytic (classical oncocytic, follicular oncocytic, and Warthin-like) variant PTC (*n* = 18), and aggressive (tall cell, hobnail, solid/trabecular, and diffuse sclerosing) variant PTC (*n* = 9). The primary tumor was not available for revision in three cases.

Our cohort constituted 202 (83.8%) females and 39 (16.2%) males, the mean age of the patients was 51.7 ± 15.2 years. Gender distribution did not reveal differences between histological subtypes. Patients presenting with aggressive variant PTC were older (mean 57.6 ± 14.8 years).

The most frequent type of surgery was total thyroidectomy (*n* = 228, 94.6%). Lymphadenectomy was performed in 28 (11.6%) patients. Patients presenting with aggressive variant PTC and classical PTC were more frequently submitted to associated lymphadenectomy (*n* = 3, 33.3% and *n* = 20, 12.7%, respectively) than the remaining patient population (*p* = 0.035). 

Median tumor size was 12.0 ± 10.0 mm, and 97 (40.2%) were mPTC. The presence of solid components was a rare event (*n* = 19, 8.0%), more often present in aggressive variant PTC (*n* = 3, 33.3%, *p* < 0.001). Inflammatory infiltrate was a frequent finding (*n* = 212, 89.5%) that was present in all aggressive variant PTC (*n* = 9, *p* = 0.005). PBs appeared in 82 (34.6%) tumors, they predominated in classical PTC and aggressive variant PTC (*n* = 72, 46.2% and *n* = 3, 33.3%, respectively), with statistically significant differences (*p* < 0.001). Dystrophic calcification (DC) was found in 57 (23.9%) and necrosis in five (2.1%) tumors.

The majority of tumors were unilateral (*n* = 199, 83.3%), unifocal (*n* = 151, 62.7%), and with an expansive growth pattern (*n* = 133, 55.6%). Lymph vessel invasion was present in 47 tumors (19.8%). Classical PTC (*n* = 36, 23.1%) and aggressive variant PTC (*n* = 4, 44.4%) evidenced a higher occurrence of lymph vessel invasion (*p* = 0.023). Venous invasion was a rare event (*n* = 16, 6.7%) that was more often in follicular variant PTC and oncocytic variant PTC (*n* = 8, 4.5% and, *n* = 3, 16.7%, respectively; *p* = 0.003).

Most tumors (*n* = 168, 70.6%) were located at the gland periphery or contacted the gland capsule, but only 96 (41.7%) tumors extended beyond the capsule. Histological extension to the perithyroidal soft tissue was present in 96 (41.7%) tumors. Gross ETE was present in 10 (4.2%) patients, three (33.3%) had aggressive variant PTC (*p* = 0.009). Resection margins were incomplete in 31 (12.9%) patients.

The histological examination of the thyroid gland revealed associated lesions in 158 (65.8%) patients. Multinodular goiter and lymphocytic thyroiditis were the most common lesions found (*n* = 63, 39.9%, and *n* = 79, 50.0%, respectively).

### 2.2. Post-Operative Staging and Radioiodine (RAI) 

There were 193 (80.0%) tumors smaller than 20mm. Cervical LNMs were present in 34 (14.1%) patients. Despite not reaching statistical significance, classical PTC and aggressive variant PTC had higher proportions of tumors with LNMs (*n* = 25, 15.9% and *n* = 2, 22.2%, respectively). Central LNMs were present in 18 (7.5%) patients, and lateral LNMs were present in 16 (6.6%) patients. Extranodal disease extension was present in eight (44.4%) of the central LNMs and five (31.2%) of the lateral LNMs.

DMs were present at diagnosis in four (1.7%) patients, two cases of classical PTC and two cases of follicular variant PTC. The majority of our population were stage I (*n* = 225, 93.4%), there were 12 (5.0%) stage II patients and four (1.7%) stage IVB patients.

RAI treatment was performed in 182 (75.5%) patients, 26 (14.3%) patients were submitted to more than one treatment. The median RAI dose administered was 100.0 ± 28.0 mCi (0–740 mCi).

We subdivided our cohort into two groups, depending on whether they were submitted to RAI. In the RAI group (*n* = 182), half of patients (*n* = 96) had intermediate risk of recurrence. The majority (*n* = 44, 74.6%) of non-RAI-treated patients (*n* = 59) had low risk of recurrence.

### 2.3. Follow-Up

The median follow-up was 71.8 ± 40.4 months (2.1–197.4 months).

We maintained the two group subdivision during follow-up evaluation, since Tg (thyroglobulin) levels are different concerning the type of surgery and RAI administration.

The first dynamic risk stratification (FDRS) analysis was made 24 months after treatment, as proposed by Tuttle et al. [11]. At that time, 118 (68.2%) RAI treated patients revealed an excellent response to treatment. Nineteen (11.0%) patients had a structural incomplete response. At FDRS, 41 (71.9%) of the non-RAI-treated patients had an excellent response to treatment.

At the end of follow-up, 14 (7.7%) RAI-treated patients had a structural incomplete response. Most non-RAI-treated patients had excellent or indeterminate response to treatment (*n* = 56, 96.5%), none had structural incomplete response or needed to be submitted to further treatment.

### 2.4. Association Between Clinicopathological Features and Recurrent/Persistent Disease

In our series, recurrence was observed in 12 (5.0%) patients. Persistence was noticed in 45 (18.7%) patients. A detailed presentation of data can be accessed in Table S3.

**Figure 1:**
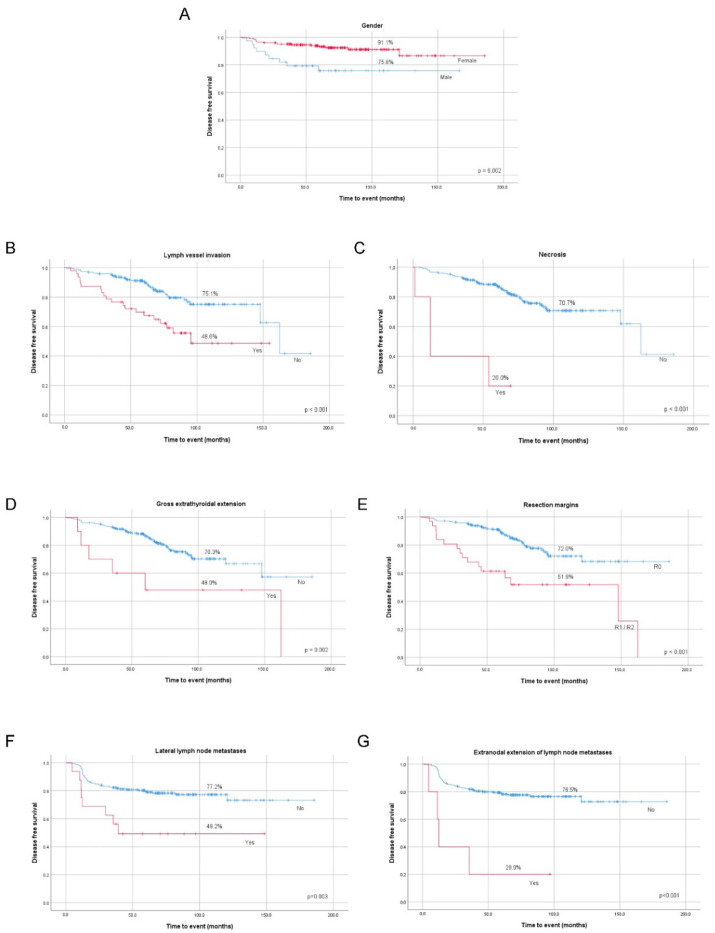
Kaplan–Meier curves of papillary thyroid cancer (PTC) recurrent/persistent disease status according to gender (**A**), lymph vessel invasion (**B**), necrosis (**C**), gross extrathyroidal extension (**D**) and resection margins (**E**), metastasized lateral lymph nodes (**F**), and extranodal extension of metastasized lateral lymph nodes (**G**).

We found a significant association between increased risk of recurrent/persistent disease and male gender, lymph vessel invasion, presence of necrosis, gross ETE, incomplete surgical resection, lateral compartment LNM, and extranodal extension of lateral LNM in the whole group (Table 1). All the clinicopathological features mentioned above significantly lowered DFS at 10-year follow-up (Figure 1A–G).

In classical PTC, a significant association was found between increased risk of recurrent/persistent disease and larger tumor size, lymph vessel invasion, presence of necrosis, incomplete surgical resection, and extranodal extension of lateral LNM.

Patients with recurrent/persistent disease more often needed further RAI treatment (*p* < 0.001).

The multivariate logistic regression analysis after adjustment for age at diagnosis demonstrated that male gender (OR = 2.9 (95% CI 1.27–5.83)), lymph vessel invasion (OR = 2.8 (95% CI 1.34–5.83)), and incomplete surgical resection (OR = 4.6 (95% CI 1.98–10.67)) were independent risk factors for recurrent/persistent disease (Table 2).

### 2.5. Association Between Clinicopathological Features and Recurrent/Persistent Disease, in Stage I Patients

Considering that 225 (93.5%) patients in our series were at stage I, according to the AJCC/UICC (American Joint Committee on Cancer/Union for International Cancer Control), and that this is the stage that fits the majority of PTC patients, we performed the above analysis in this subgroup of patients.

We found a significant association between increased risk of recurrent/persistent disease and nodule size, lymph vessel invasion, gross ETE, and incomplete surgical resection.

The multivariate logistic regression analysis after adjustment for age and gender at diagnosis demonstrated that gender (OR = 2.6 (95% CI 1.05–6.22); *p* = 0.039), lymph vessel invasion (OR = 2.9 (95% CI 1.33–6.31); *p* = 0.007), and incomplete surgical resection (OR = 4.4 (95% CI 1.83–10.70); *p* = 0.001) remained independent risk factors for recurrent/persistent disease in stage I patients.

### 2.6. Association Between Clinicopathological Features and Structural Disease Status

A detailed presentation of data can be accessed in Table S4.

**Figure 2:**
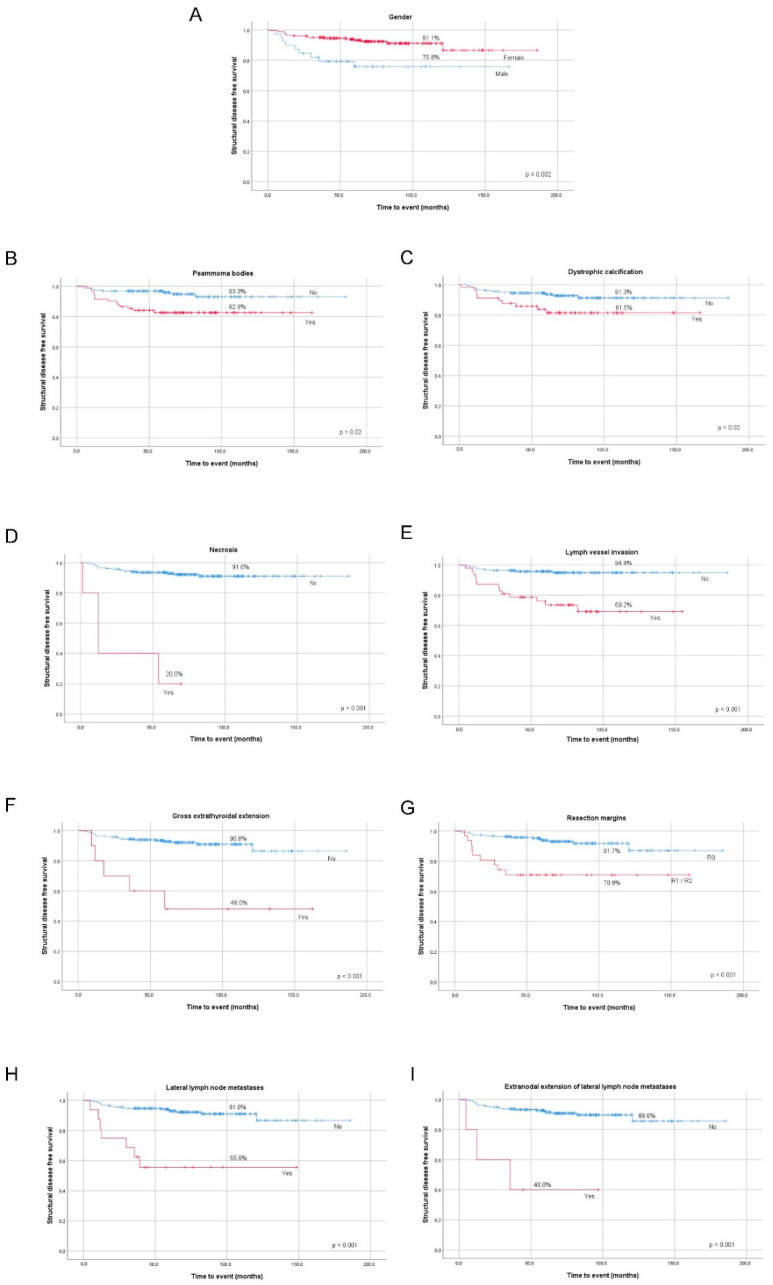
Kaplan–Meier curves of PTC structural disease status by gender (**A**), presence of psammoma bodies (**B**), presence of dystrophic calcification (**C**), presence of necrosis (**D**), lymph vessel invasion (**E**), gross extrathyroidal extension (**F**), resection margins (**G**), presence of metastasized lateral lymph nodes (**H**), and extranodal extension of metastasized lateral lymph nodes (**I**).

We found a significant association between increased risk of structural disease status and male gender, tumors bigger than 10mm, presence of PBs, DC, necrosis, lymph vessel invasion, gross ETE, incomplete surgical resection, lateral compartment LNM, and extranodal extension of lateral LNM in the whole group (Table 3). All the clinicopathological features mentioned above significantly lowered DFS at 10-year follow-up (Figure 2A–I).

In classical PTC, a significant association was found between increased risk of structural disease status and presence of PBs and DC, lymph vessel invasion, incomplete surgical resection, and lateral compartment LNM.

The multivariate logistic regression analysis after adjustment for age at diagnosis and gender demonstrated that PBs (OR = 4.5 (95% CI 1.49–13.45)), lymph vessel invasion (OR = 6.9 (95% CI 2.47–19.22)), and gross ETE (OR = 16.1 (95% CI 3.30–78.68)) are independent risk factors for structural disease status (Table 4).

### 2.7. Association Between Clinicopathological Features and Structural Disease Status, in Stage I Patients

In stage I patients, we found a significant association between increased risk of structural disease status and nodule size, presence of PBs, DC, lymph vessel invasion, gross ETE, incomplete surgical resection, and lateral compartment LNM in the whole group.

The Cox regression analysis after adjustment for age at diagnosis and gender demonstrated that PBs (OR = 4.86 (95% CI 1.37–17.30); *p* = 0.015), lymph vessel invasion (OR = 7.76 (95% CI 2.35–25.65); *p* = 0.001), and gross ETE (OR = 24.13 (95% CI 3.55–163.91); *p* = 0.001) are independent risk factors for structural disease status in stage I patients.

### 2.8. Association Between Clinicopathological Features and Disease-Specific Mortality

A detailed presentation of data can be accessed in Table S5. 

**Figure 3:**
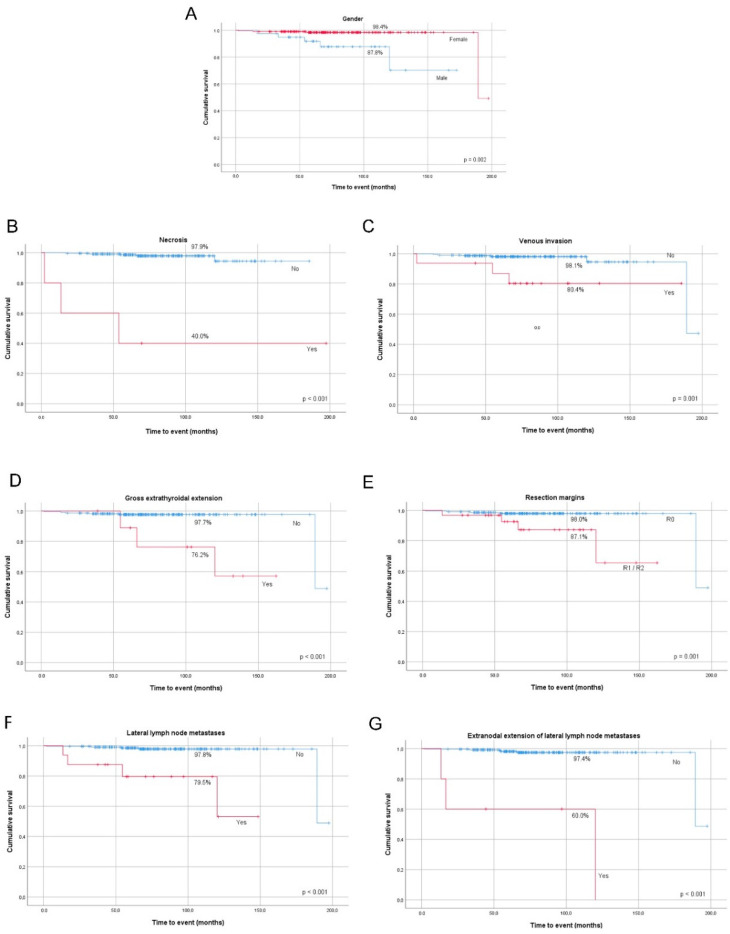
Kaplan–Meier survival curves of PTC disease-specific survival (DSS) by gender (**A**), presence of necrosis (**B**), venous invasion (**C**), gross extrathyroidal extension (**D**) resection margins status (**E**), metastases to lateral lymph nodes (**F**), and extranodal extension of lateral lymph node metastases (**G**).

A significant association was found between increased risk of DSM (disease-specific mortality) and older age at diagnosis, male gender, larger tumors, presence of necrosis, lymph vessel invasion, venous invasion, gross ETE, lateral compartment LNM, and extranodal extension of lateral LNM in the whole group (Table 5). All the clinicopathological features mentioned above significantly lowered DSS at 10-year follow-up (Figure 3A–G).

In classical PTC, a significant association was found between increased risk of DSM and older age and larger tumors. In follicular variant PTC, a significant association was found between increased risk of DSM and venous invasion. DSM events were not observed in oncocytic variant PTC.

All patients that had DSM were treated with RAI. All of them had disease persistence and received higher RAI doses (*p* < 0.001). They either had DMs at presentation or (the majority) developed DMs (*p* < 0.001) during follow-up.

The multivariate logistic regression analysis after adjustment for age at diagnosis demonstrated that gender (HR = 15.4 (95% CI 1.69–140.84)), venous invasion (HR = 16.7 (95% CI 1.50–185.60)), and lateral compartment LNM (HR = 26.7 (95% CI 2.88–246.87)) are independent risk factors for DSM (Table 6).

## 3. Discussion

Thyroid cancer is a disease carrying a good or very good prognosis. However, recurrent/persistent disease can be present in a substantial proportion of these patients and there is lack of effective indicators that can be used in the daily clinical practice to stratify patients. In most cases, clinicians have difficulty separating a low risk disease from patients at risk of persistence/progression/death.

Our study was done in a central hospital, based upon a series of consecutive patients (more than 80% of the patients were middle-aged women) submitted to thyroid surgery between January 2002 and December 2015. All the patients had a diagnosis of PTC, without selection bias other than the inclusion/exclusion criteria.

At 10-year follow-up, our cohort of patients displayed an overall survival of 96.3%, with a recurrent/persistent disease rate of 23.7% (in accordance with the literature) [6,12]. Seventy-eight percent of the patients did not have evidence of disease at the end of follow-up. Most patients presented stage I or II disease. The above data indicate that our PTC series is composed of low-risk cases and is a reliable series to study the prognosis influence of the clinicopathological features, in an unbiased daily clinical setting.

Total thyroidectomy and RAI ablation were the most frequent treatments for PTC patients. In 2015, ATA (American Thyroid Association) guidelines [2] emphasized the role of lobectomy and isthmectomy and limited the RAI role in PTC treatment, but these guidelines were not used in our cohort. The patients who were treated without receiving RAI had excellent outcomes, supporting observations that in selected cases, RAI is not needed to assure definitive PTC treatment [13]. 

Previous studies attributed to male gender a significant role in worse PTC prognosis [14,15]. In our series, male patients displayed a higher risk of persistent/recurrent disease and of DSM. Our results confirm male gender as an independent prognostic factor, affecting recurrent/persistent DFS and DSM [10].

An association between matrix calcification and thyroid malignancy has been previously proposed [3]. Older patients will have increased tumor size and more frequent calcifications and this may explain why tumors with calcifications are associated with more aggressive features [12]. In our work, we evaluated the presence of DC and PBs. The presence of PBs may be seen either within PTC or in adjacent thyroid tissue [16], which is useful for diagnosing classical PTC, but whether or not the presence of PBs is associated to PTC prognosis [3,17,18,19,20] still remains controversial.

The aforementioned controversy stems from the fact that PBs are frequently found in mPTC [18] and associated with the indolent course of this type of cancer [19]. We have described a significant correlation between PTC with PBs and younger patients, ETE, and LNM [3,20] and other authors also reported a significant association with high stage grouping (stage IVa) and poorer disease-free survival [17]. In our series, the presence of PBs was an independent factor for structural disease status in the multivariate analysis. Our result indicates, for the first time, a relevant role of PBs as an indicator of patient outcome, providing one rules out the mPTC. 

The presence of PBs can be revealed by a careful ultrasonography, because there is a strong association between PBs and microcalcifications [12,17,20]. In fine needle aspiration cytology, PBs are an excellent marker of PTC diagnosis [3]. Pre-operative knowledge of PBs’ relation to microcalcifications and to structural disease status, observed in our series, can provide meaningful information in surgeons’ decision-making processes.

A well-known factor associated with an adverse outcome regardless of architectural pattern or nuclear features is necrosis [21]. The presence of necrosis increased the risk for all the evaluated outcomes. Although present in five cases, the prognosis importance of such a finding (necrosis) was so high that we had to remove it from the multivariate analysis; otherwise, necrosis would be the only significantly predictive factor for any outcome. 

The impact of lymph vessel invasion and venous invasion on tumor behaviour is recognized by the ATA risk stratification system [2]. Taking in consideration the above, we realize the importance of analyzing lymph vessel invasion and venous invasion in patient outcome. We performed this analysis in our series, despite recognizing the difficulty and limitation to distinguishing venous invasion from lymph vessel invasion by H&E (Hematoxylin and eosin) studies alone [21,22]. In the multivariate analysis, lymph vessel invasion remained an independent prognostic factor for recurrent/persistent disease and structural disease status. In multivariate analysis, venous invasion is associated with DSM. 

This issue has recently been emphasized by the International Collaboration on Cancer Reporting (ICCR). The dataset developed by the ICCR for the pathology reporting of thyroid resection specimens for carcinoma declares that it is mandatory to specifically report on the status of venous invasion in PTC [23].

Tumor location in the thyroid gland is suggested by some authors to predispose to ETE, since superficial tumor location may facilitate tumor spread into lymphatic channels and regional lymph nodes [24]. We did not find a significant association between tumor location or minimal ETE and any of the outcomes. This observation fits with other authors that stated that minimal ETE is not an independent prognostic factor for DFS or DSS [21,25], and is associated with a low risk (3–9%) of recurrence [26]. Our result also supports the recent revision and downgrading of staging in cases with minimal ETE [2].

Gross ETE is described by some authors as an independent prognostic factor, affecting patients’ DFS and DSS [27]. Other authors suggested that gross ETE particularly affects DFS of patients with tumors measuring >20 mm [28]. In our series, in which 80% of the tumors were <20 mm, gross ETE had a deleterious impact on patients’ evaluated outcomes. In the multivariate analysis, gross ETE remained a significant independent factor for structural disease status. This observation fits with other authors that described gross ETE association with an increased recurrence risk (23–40%) [21,26] being a major predictor for poor outcome in PTC [25].

The assessment of ETE is crucial in pre-operative staging of TC [29] to decide the extent of the surgery; if ETE is present, RAI treatment will be performed. Ultrasonography is routinely done in every thyroid patient and has high sensitivity for assessing gross ETE [29]. Given the risk of gross ETE for structural disease observed in our series, pre-operative knowledge can be of major significance in surgeons’ decision-making processes.

Having usually an indolent growth and excellent prognosis, the quality of surgery is pivotal in PTC treatment [30]. The surgeon is an important part of a personalized approach and a cornerstone involving the patient, the clinician, and the pathologist. A detailed surgical report is crucial for proper staging and further treatment and incomplete surgical resection is a high risk factor for recurrence, as well as DSM [11,25]. In our series, incomplete surgical resection was reported in 12.9% patients (in accordance with the literature [31]), and remained a significant independent prognostic feature for recurrent/persistent disease in the multivariate analysis. 

Cervical LNM is a common finding in PTC, affecting 20% to 80% of patients [20,21]. Some authors state that clinically evident cervical LNM seems to be the fundamental indicator in terms of prognosis [32]. PTC frequently recurs in regional lymph nodes [28] and cervical LNM, at initial surgery, increases the incidence of recurrent disease during follow-up [30,32] and decreases DSS in some studies. Controversy exists regarding appropriate surgical management of cervical lymph nodes without evidence of metastases. Some authors prefer prophylactic central compartment lymphadenectomy because second surgery for recurrence in the central compartment has a greater risk of recurrent laryngeal nerve injury and permanent hypoparathyroidism. Ito et al. highlighted that a large number of central LNMs (>5), not just their presence, has an independent prognostic impact [33]. Other authors have emphasized that prophylactic central lymphadenectomy does not improve patients’ outcomes [34]. ATA recommends prophylactic central lymphadenectomy in the presence of advanced tumors and lateral compartment LNM [2].

In our series, central LNM was not associated with patient outcome. The absence of an association between central LNM and prognosis can be related to the tumors’ small size (only 4/18 tumors were ≥21 mm) or the small number of central LNMs (13/18 tumors had fewer than five LNMs). The absence of an association between central LNM and prognosis can also indicate that central LNM has no significant association with patient prognosis. In contrast, we observed a significant impact of lateral compartment cervical LNM and of extranodal extension of lateral LNM in all evaluated outcomes, as is described in the literature [30]. In the multivariate analysis, only the presence of lateral compartment LNM remained a significant independent prognostic feature, increasing the risk of DSM. 

Ultrasonography evaluation of lateral LNM is mandatory in pre-operative staging of thyroid cancer to plan the surgery extension. In our series, we observed a significant risk of lateral LNM for DSM and confirmed its pre-operative importance in surgeons’ decision-making processes.

The overall good prognosis of PTC is expressed in patients’ long-term survival and in the fact that most PTC patients are stage I, according to the AJCC/UICC, at diagnosis. In our series, 225 (93.5%) patients were stage I at diagnosis. Our results are of even greater importance since the same clinicopathological factors remained as statistically significant outcome predictors when statistical analysis, for recurrence/persistent disease and structural disease, was performed when restricted to stage I patients.

## 4. Materials and Methods 

### 4.1. Patient Cohort and Tissue Samples

Each file of 1652 consecutive patients, with thyroid tumors, submitted to thyroid surgery at the central hospital of Vila Nova de Gaia/Espinho (CHVNG/E), from January 2002 to December 2015, was reviewed by a single medical doctor (A.A.P.). TC was diagnosed in 461 (26%) patients. PTC was present in 423 (91.8%) of them. We excluded 158 patients: 29 patients had tumors smaller than 2 mm, 16 patients had not enough data and/or follow-up, and 113 patients had no material available to be rescued. In the present series (*n* = 265), we diagnosed 24 non-invasive follicular thyroid neoplasms with papillary-like nuclear features, which were excluded, following the recommendations of the World Health Organization (WHO) [7]. Inclusion criteria were the diagnosis of PTC in patients older than 18 years, followed for a minimum of two years (unless recurrence or disease-specific mortality occurred earlier), in which there were thyroid specimens for histological re-evaluation (*n* = 241). 

Formalin-fixed paraffin-embedded (FFPE) material was collected, and all tumor samples were reviewed by a single pathologist, M.R.B., according to the WHO classification, 4th edition [7]. All doubtful tumor samples were reviewed by two senior pathologists (M.S.S., M.R.B.) for consensual diagnosis. Each patient was staged according to the 8th edition AJCC/UICC staging system [35]. Histological criteria adopted are summarized in Table S1.

All procedures described in this study were in accordance with national and institutional ethical standards. The study was conducted in accordance with the Declaration of Helsinki, and the protocol was approved by the Ethics Committee of CHVNG/E (Project investigation 30/2016, 2016-01-28, Comissão de Ética do Centro Hospitalar de Vila Nova de Gaia/Espinho). According to Portuguese law, informed consent is not required for retrospective studies.

### 4.2. Patient Follow-Up

PTC patients underwent surgery with or without RAI treatment. Surgical options depended on tumor characteristics (size, ETE, clinically identified LNM, DM), and patient’s age and gender. Lymphadenectomy was performed whenever LNMs were found before surgery or intra-operatively. RAI treatment was dependent on type of surgery performed (total vs. lobectomylobectomy and isthmectomy, surgery completeness), post-surgery pathological findings (size, ETE, lymph vessel and venous invasion, LNM), presence of DM, and patient’s data.

RAI therapy was given 4 weeks after levothyroxine (LT4) withdrawal or using a recombinant thyroid stimulating hormone (rhTSH) preparation. This treatment was followed by post-therapy scintigraphy seven days later. Stimulated thyroglobulin (sTg) was evaluated at the time of RAI therapy and it was defined as the thyroglobulin (Tg) level measured when serum TSH was >30 mIU/L. The post-ablation Tg level was assessed approximately 12 months after surgery.

During the first follow-up year, patients were followed every three months and, in subsequent years, twice a year. Serum biochemical evaluation (TSH, free LT4, Tg, anti-Tg antibodies (TgAb)) and neck ultrasonography (US) were routinely performed. Computed tomography (CT) scans, 18-fluorodeoxyglucose positron emission tomography (18-FDG-PET) scans or scintigraphy were performed only if previous exams were doubtful or suspicious. During follow-up, we used the 2015 ATA risk stratification system [2] to predict the risk of recurrent or persistent disease, in patients who were treated with RAI. In non-RAI-treated patients, we used the risk stratification system for patients treated without RAI published by Momesso et al. [36]. All patients were stratified as having four levels of possible response to treatment: excellent, indeterminate, biochemically incomplete, and structurally incomplete [2,36].

Structural disease status was detected by appropriate imaging modalities and, whenever possible, confirmed by cytology. Additional surgery was performed whenever feasible. If disease was unresectable, patients received RAI therapy. One patient with RAI-refractory disease was submitted to tyrosine kinase inhibitor treatment.

### 4.3. Clinical Endpoints

No clinical evidence of disease (NED) at final follow-up was established if patients had Tg levels that fit an excellent response [2], no detectable TgAb, and no structural evidence of disease. Patients were classified as having persistent disease whenever Tg values fit an indeterminate or incomplete response (elevated basal or sTg values alone, without structural correlation), or there was any evidence of disease on cross-sectional imaging (US, CT scan), functional imaging (RAI scan or 18-FDG-PET scan), or biopsy-proven disease (cytology or histology). 

Patients were considered to have a positive structural disease status if any of the following conditions were met: (1) positive cytology/histology, (2) highly suspicious lymph nodes or thyroid bed nodules in the neck US (hypervascularity, cystic areas, heterogeneous content, rounded shape, or enlargement on the follow-up), (3) findings in scintigraphy, 18-FDG-PET scans, or other cross-sectional imaging highly suspicious for metastatic disease. Recurrence was defined if new biochemical, structural, and/or functional evidence of disease was detected following any period of NED. Recurrent/persistent disease (*n* = 57) was considered if patients had indeterminate or incomplete response to treatment, whether disease was only biochemical (*n* = 33) or biochemical and structural (*n* = 24). Disease-specific mortality (DSM) was also an endpoint (patients dying of unrelated conditions had the final status determined based on data available before their demise). 

### 4.4. Statistical Analysis

Statistical analysis was performed with IBM SPSS Statistics version 25 (IBM, New York, NY, USA). The results were expressed in absolute frequency, percentage, mean ± standard deviation (Std), or median ± interquartile range (IR). Univariate analysis and correlation between recurrent/persistent disease, structural disease status, DSM, and clinicopathological features (age, gender, tumor size, ETE, lymph vessel invasion, venous invasion, for instance) were performed using a chi-squared test, Fisher’s exact test, and logistic regression analysis. Unpaired *t*-tests or Mann–Whitney tests were applied when adequate. Multivariate analysis was performed using Cox regression. In the regression models, all the features significantly associated with each specified outcome in the univariate analysis were included in the multivariate analysis. The model was adjusted for gender and age at the time of surgery. Results were considered statistically significant at a *p* value < 0.05. 

## 5. Conclusions

Our study was performed in a consecutive series of patients submitted to surgery in a single institution, in a defined period, using clinical data collected by a single medical doctor and tumors reviewed by a single pathologist. The patients were followed for a median of 72 months. With this methodology, we achieved an unbiased population from a single institution, and it was rewarding to realize that we observed an excellent prognosis and low mortality rates as major conclusions points. 

Having been referred to as predictors of poor outcome in previous studies, the evaluated features had not been previously addressed in a systematic way in relation to patient outcomes (recurrent/persistent disease, structural disease status, and disease-specific mortality), as far as we are aware. Our study supports that both pre-surgical factors, such as male gender, presence of PBs, gross ETE, and lateral compartment LNM, as well as lymph vessel invasion, venous invasion, presence of necrosis, and incomplete surgical resection, should be taken into consideration regarding treatment and follow-up of PTC patients. Our study emphasizes the important role of complete resection margins and careful and systematic lymphadenectomy in the presence of lateral compartment LNM.

## Figures and Tables

**Table 1:** Univariate analysis regarding recurrent/persistent disease.

Clinicopathological Features	Univariate Analysis	
	OR (95% CI)	*p*-Value
Gender	2.732 (1.324–5.637)	0.007
Necrosis	14.560 (1.592–133.196)	0.018
Lymph vessel invasion	3.399 (1.710–6.756)	<0.001
Gross extrathyroidal extension	5.400 (1.467–19.881)	0.011
Resection margins	4. 507 (2.057–9.872)	<0.001
Lateral lymph node metastases	3.592 (1.283–10.059)	0.015
Extranodal extension of lateral lymph node metastases	13.811 (1.511–126.219)	0.020

**Table 2:** Multivariate logistic regression analysis regarding recurrent/persistent disease.

Clinicopathological Features	Multivariate Analysis	
	OR (95% CI)	*p*-Value
Gender	2.870 (1.273–5.833)	0.011
Lymph vessel invasion	2.795 (1.340–5.833)	0.006
Resection margins	4.596 (1.980–10.671)	<0.001

**Table 3:** Univariate analysis regarding structural disease status.

Clinicopathological Features	Univariate Analysis	
	OR (95% CI)	*p*-Value
Gender	3.487 (1.413–8.605)	0.007
Nodule size (cut-off 10 mm)	2.968 (1.074–8.201)	0.036
Psammoma bodies	3.783 (1.515–9.445)	0.004
Dystrophic calcification	2.750 (1.134–6.666)	0.025
Necrosis	47.556 (5.045–448.261)	0.001
Lymph vessel invasion	7.690 (3.048–19.402)	<0.001
Gross extrathyroidal extension	11.105 (2.950–41.801)	<0.001
Resection margins	5.291 (2.074–13.500)	<0.001
Lateral lymph node metastases	8.944 (2.981–26.841)	<0.001
Extranodal extension of lateral lymph node metastases	14.591 (2.312–92.076)	0.004

**Table 4:** Multivariate logistic regression analysis regarding structural disease status.

Clinicopathological Features	Multivariate Analysis	
	OR (95% CI)	*p*-Value
Psammoma bodies	4.478 (1.491–13.451)	0.008
Lymph vessel invasion	6.887 (2.467–19.224)	<0.001
Gross extrathyroidal extension	16.118 (3.302–78.678)	0.001

**Table 5:** Univariate analysis regarding PTC disease-specific mortality.

Clinicopathological Features	Univariate Analysis	
	HR (95% CI)	*p*-Value
Age	1.067 (1.012–1.124)	0.016
Gender	7.279 (1.861–24.481)	0.004
Nodule size	1.079 (1.035–1.124)	<0.001
Necrosis	68.100 (9.248–501.489)	<0.001
Gross extrathyroidal extension	16.000 (3.306–77.440)	0.001
Resection margins	6.044 (1.529–23.899)	0.010
Lymph vessel invasion	7.421 (1.706–32.273)	0.008
Venous invasion	8.346 (1.872–37.205)	0.005
Lateral lymph node metastases	14.667 (3.484–61.745)	<0.001
Extranodal extension of metastasized lateral lymph nodes	57.500 (8.065–409.938)	<0.001

**Table 6:** Multivariate logistic regression analysis regarding PTC-specific mortality.

Clinicopathological Features	Multivariate Analysis	
	HR (95% CI)	*p*-Value
Age	1.077 (1.010–1.148)	0.023
Gender	15.426 (1.690–140.844)	0.015
Venous invasion	16.667 (1.497–185.606)	0.022
Lateral lymph node metastases	26.686 (2.885–246.870)	0.004

The analysis was not done for stage I patients since only three patients had disease-specific mortality (DSM).

## Supplementary Materials

The following are available online at https://www.mdpi.com/2072-6694/12/11/3186/s1, Table S1: Histopathological variables and considered criteria, Table S2: Clinicopathological features, Table S3: Clinicopathological features concerning recurrent/persistent disease, Table S4: Clinicopathological features concerning structural disease status, Table S5: Clinicopathological features concerning PTC-specific mortality. The graphical abstract was created with Biorender.com.
