# Supplementary material for: Clinicopathological Features as Prognostic Predictors of Poor Outcome in Papillary Thyroid Carcinoma
Source: Cancers (Basel). 2020 Oct 29;12(11):3186. doi: 10.3390/cancers12113186 (PMC7693726; doi:10.3390/cancers12113186)
Supplement: Supplementary file 1 [file cancers-12-03186-s001.zip › cancers-968309-supple-xml/cancers-968309-supple-xml.pdf]

Supplementary Materials: Clinicopathological Features as Prognostic Predictors of Poor Outcome in Papillary Thyroid Carcinoma

Antónia Afonso Póvoa, Elisabete Teixeira, Maria Rosa Bella-Cueto, Miguel Melo, Maria João Oliveira, Manuel Sobrinho-Simões, Jorge Maciel and Paula Soares

Table S1. Histopathological variables and considered criteria.

Analyzed Category	Criteria	Evaluation
Poorly differentiated component	percentage within the primary tumor	%
Oncocytic component	polygonal shape cells, with large (three times the nucleus) eosinophilic and granular cytoplasm	%
Solid component	compact cell distribution or in trabecular disposition, without follicle formation	%
Papillae	papillary architecture, with neoplastic cells covering fibrovascular stalks	%
Inflammatory infiltrate	may range from only few isolated lymphocytes within the tumor or in its periphery, always less than 10 cells aggregated, to–large lymphocytes groups that occupy at least 20% of the tumor	Absent/present
Fibrosis	all kinds: sclerosing, desmoplastic, hyaline tracts, central post ischemic, central hyalinization, wide fibrovascular stalks	%
Psammoma bodies	when present, even if it's just one	Absent/present
Dystrophic calcification	irregular shape calcification, whose size is bigger than psammoma bodies, no spherical or concentric shape	Absent/present
Necrosis	presence of focal ischemic or lytic necrosis	Absent/present
Tumor peripheric location	a. A–tumor is close (1–3 mm) to thyroid capsule without reaching it and 50% of the tumor is located more than 10mm of the thyroid capsule	A
	b. B-tumor contacts thyroid capsule or is close to it (1–3mm) and 50% of the tumor is located less than 10 mm of the thyroid capsule	B
	c. No–tumor is located at more than 3 mm of thyroid capsule	C
Tumor capsule invasion	tumor cells beyond tumor capsule<br>a. Minimal–or less invasive foci	Minimal/Extensive

b. Extensive–more than 3 invasive foci

Analyzed category	Criteria	Evaluation
Tumor growth	a. Expansive–more than 95% of the tumor has regular limit<br>b. Infiltrative–more than 5% of the tumor has infiltrative border	Expansive/Infiltrative
Extra-thyroidal extension	tumor cells beyond thyroid capsule<br>a. Minimal–affects fibroadipose tissue with prominent vessels or nerves surrounding thyroid gland<br>b. Gross–affects strap muscles	Minimal/ Gross
Lymphatic invasion	a. Yes–mesothelial cover of the invaded vessel	Yes
	b. Doubtful–round structure apart from the tumor, in relation with artery or vein, or that forms a longitudinal path inside the thyroid gland apart from the tumor	No
	c. No?	Doubtful
Venous invasion	1.venous invasion of tumor capsule vessels or outside the thyroid capsule	Absent/present
NIFTP	capsulated or well delimitated tumor, follicular patterned, with papillary-like nucleus, without capsular or vascular invasion and without solid/trabecular/insular pattern	Absent/present

© 2020 by the authors. Licensee MDPI, Basel, Switzerland. This article is an open access article distributed under the terms and conditions of the Creative Commons Attribution (CC BY) license (http://creativecommons.org/licenses/by/4.0/).
